# Locus specific reduction of L1 expression in the cortices of individuals with amyotrophic lateral sclerosis

**DOI:** 10.1186/s13041-022-00914-x

**Published:** 2022-03-28

**Authors:** Abigail L. Pfaff, Vivien J. Bubb, John P. Quinn, Sulev Koks

**Affiliations:** 1 Perron Institute for Neurological and Translational Science, Perth, 8 Verdun Street, Nedlands, WA 6009 Australia; 2 Centre for Molecular Medicine and Innovative Therapeutics, Murdoch University, Perth, WA Australia; 3 Department of Pharmacology and Therapeutics, Institute of Systems, Molecular and Integrative Biology, University of Liverpool, Liverpool, UK

**Keywords:** Amyotrophic lateral sclerosis, Long interspersed element-1 (LINE-1/L1), Retrotransposon, Expression

## Abstract

The activation and dysregulation of retrotransposons has been identified in the CNS of individuals with the fatal neurodegenerative disorder Amyotrophic lateral sclerosis (ALS). This includes elements from multiple different families and subfamilies of retrotransposons, however there is limited knowledge of the specific loci from which this expression occurs in ALS. The long interspersed element-1 (L1) is the only autonomous retrotransposon in the human genome and members of this family of elements maintain the ability to mobilise. Despite L1s contributing to 17% of the human genome only 80–100 L1s encode the required proteins for mobilisation and are retrotransposition competent. Identifying the specific loci from which L1 expression occurs will inform on the potential functional consequences of their expression, such as the potential for somatic retrotransposition or DNA damage caused by the endonuclease activity of the ORF2 protein of the L1. Here we characterised L1 loci expression using the L1EM tool (https://github.com/FenyoLab/L1EM) in RNA sequencing data from 518 samples across four tissues (motor cortex, frontal cortex, cerebellum and cervical spinal cord) in the Target ALS cohort obtained from the New York Genome Center. There was a significant reduction in total intact L1 expression (those that encode functional proteins) in two brain regions of individuals with ALS compared to controls and clustering of the ALS brain regions occurred based on their intact L1 expression profile. Although overall the levels of L1 expression were reduced in ALS/ALS with other neurological disorder (ND) there were individuals in which L1s were expressed at much higher levels than the rest of the ALS/ALSND cohort. Expressed L1 loci were more frequently located in introns compared to those not expressed and the level of L1 expression positively correlated with the expression of the gene in which it was located. Significant differences were observed in the expression profiles of L1s in ALS and specific features of these elements, such as location in the genome and whether or not they are intact, were significantly associated with those that were expressed in the cohort.

**Supplementary Information:**

The online version contains supplementary material available at 10.1186/s13041-022-00914-x.

## Introduction

Amyotrophic lateral sclerosis (ALS) is a rare progressive neurodegenerative disease resulting in the loss of motor neurons in the brain and spinal cord. Death usually occurs within 3–5 years of symptom onset and there are currently no treatments to cure or prevent the disease from progressing further. The identification of pathogenic variants in genes such as *SOD1*, *FUS*, *TARDBP* and *C9ORF72* and a range of additional genetic variants associated with disease risk have informed on the mechanisms of ALS development [1]. Several processes, such as oxidative stress, mitochondrial dysfunction, protein aggregation, inflammation and RNA processing and toxicity, are thought to be involved in disease pathogenesis [2]. However, the exact causation of the neurodegeneration occurring in ALS is still to be determined. One area of study aiming to understand the pathogenesis of the disease further is the dysregulation and expression of retrotransposons; a type of mobile DNA contributing to nearly half of the human genome [3].

Retrotransposons are divided into two classes, those with long terminal repeats (LTRs) and those without (non-LTRs), both of which mobilise through a ‘copy and paste’ mechanism involving the reverse transcription of an RNA intermediate. Initial evidence for the potential involvement of retrotransposons in ALS was the detection of elevated reverse transcriptase activity in the sera of ALS patients compared to unaffected controls that was not attributed to an exogenous retroviral infection [4, 5]. Endogenous retroviruses are part of the LTR class of elements and elevated levels of the human endogenous retrovirus-K (HERV-K) were detected in the CNS of individuals with ALS compared to controls and a mouse model overexpressing a HERV-K protein led to motor neuron degeneration and motor dysfunction in the mice [6, 7]. Although this increased expression of HERV-K has not been observed in all studies [8, 9].

Using RNA sequencing data the expression of multiple classes and families of retrotransposons can be analysed and this approach has identified increased expression of both LTR and non-LTR elements in subsets of individuals with ALS [10–12]. Prudencio et al. analysed the expression of repetitive elements identifying an increase in several of these elements, including LTR and non-LTR retrotransposons, in the frontal cortex of individuals with ALS who were carriers of the *C9ORF72* expansion when compared to those without the expansion and healthy controls [10]. This was further confirmed by Pereira et al. [12]. Tam et al. utilised machine learning which identified three subtypes of ALS based on transcriptomic data from the frontal and motor cortices, one of which was characterised by the activation of retrotransposons and TAR DNA-binding protein 43 (TDP-43) dysfunction [11]. Cytoplasmic accumulation of TDP-43, encoded by the gene *TARDBP* in which mutations can cause ALS, is a hallmark of the majority of ALS cases [13]. There is evidence in human tissues and cell lines that TDP-43 binds to retrotransposon transcripts, which is thought to aid in the repression of these elements [14]. Analysis of the relationship of TDP-43 and transposable elements in the SHSY5Y cell line showed that TDP-43 binds to the RNA of multiple families of retrotransposons and knock-down of TDP-43 led to the upregulation of these retrotransposon targets [11]. In addition, the loss of nuclear TDP-43 from neurons results in chromatin decondensation over long interspersed element-1 (LINE/L1) elements that belong to the non-LTR class of retrotransposons [15].

L1s are the only elements that are still able to autonomously mobilise in the human genome and although there are more than 1 million L1s annotated in hg38 only 80–100 are considered retrotransposition competent (RC) [16, 17]. RC-L1s encode a functional ORF1 protein that binds RNA and an ORF2 protein with reverse transcriptase and endonuclease activity, which are required for retrotransposition [18–20]. The ability of RC-L1s to retrotranspose can be demonstrated in either a cellular retrotransposition assay or by identifying the source element for germline or somatic L1 insertions [16, 21, 22]. These features of RC-L1s along with increased L1 expression detected in certain neurological conditions has led to the hypothesis that they are involved in disease processes through multiple mechanisms such as somatic retrotransposition, the triggering of neuro-inflammation and DNA damage from the endonuclease activity of ORF2 protein [23, 24].

Our previous work focussing on highly active RC-L1s in neurodegenerative diseases has identified a reduction in the methylation of selected RC-L1s in the motor cortex of individuals with ALS compared to healthy controls [25] and found that an increased burden of these elements in the germline is associated with Parkinson’s disease risk and progression [26]. Although there have been several studies evaluating the expression profile of specific families of retrotransposons in ALS there is limited data on loci specific expression, which could provide additional insight into the potential consequences of their expression. Here we utilise a software tool called L1EM [27] to evaluate loci specific L1 Homo sapiens (L1HS) expression to identify where in the genome these elements are being expressed from. L1HS are the youngest L1 subfamily containing those elements able to mobilise, therefore we can determine if the L1s expressed encode for functional proteins and have the potential to retrotranspose. Using RNA sequencing data from the Target ALS cohort provided by the New York Genome Center (https://www.nygenome.org/) we were able to characterise the L1 expression profile in multiple tissues from individuals with or without disease and identify significant differences in L1 expression.

## Methods

### Quantifying L1HS locus specific expression using L1EM

The L1EM tool developed by McKerrow et al. (https://github.com/FenyoLab/L1EM) [27] quantifies L1 expression in a locus specific manner categorising transcripts as either passive, which are not driven by the L1 promoter and contain sequences up and downstream of the L1, or proper transcripts that only contain sequence from the annotated L1 or 3’run-on and originate from a L1 with a 5’UTR and therefore a promoter. Only transcripts are defined as those are supported by sense reads which fall entirely with the L1 and run-on transcripts are those that are in the sense orientation and are supported by reads that overlap the L1 and downstream sequence only with no reads extending upstream of the L1. As the proper L1 expression is assigned to specific L1 loci in the reference genome it can also be determined whether the resulting transcripts have the potential to encode functional L1 proteins. The L1EM tool was used to analyse RNA-sequencing data from four different tissues (motor cortex n = 107, frontal cortex n = 136, cerebellum n = 147 and spinal cord cervical n = 128) from individuals either with ALS or ALS with other neurological disorder (ALSND) and non-neurological controls (NNC) (individuals that did not have a neurological disorder) from the Target ALS cohort obtained in BAM format from the New York Genome Center (Additional file 5: Figure S1). The L1EM tool categorises five types of transcript (passive sense, passive antisense, only, run-on and antisense) and only those deemed to be proper transcripts, originating from a L1 with a 5’UTR that were classified as ‘only’ and ‘run-on’ transcripts, were taken further in our analysis. The proper transcripts belong to L1s from the L1HS subfamily and there are 400 L1HS elements in the human genome with a 5’UTR from which these proper transcript are quantified [27]. For a specific L1 element to be classified as expressed using L1EM its expression must be supported by at least 100 read pairs and is reported as FPM (read pairs per million properly aligned). Although there are some limitations for the L1EM tool, as there is potential for reads from non-reference elements to be assigned to their source element that is present in the reference genome. The L1 loci at which proper L1 expression was detected were then divided into those that were intact (encode functional L1 proteins) and those that were not using supplementary data from McKerrow et al. [27]. Logistic regression with sex, age at death and RNA integrity numbers (RIN) as covariates was used to compare the total L1 proper expression of intact and non-intact elements between ALS/ALSND and NNC with outliers removed (those with expression more than 3 standard deviations from the mean). The values of the covariates for each sample are reported in Additional file 1: Table S1. Heatmaps were generated, using heatmap.2 in R (www.R-project.org, www.rstudio.com) to compare the mean L1 loci specific expression across tissues and cases (ALS/ALSND) and controls (NNC). The mean expression of L1s that were expressed in > 25% of individuals for a particular tissue were compared between cases (ALS/ALSND) and controls (NNC) and the p values were adjusted using Benjamini–Hochberg with a 5% false discovery rate.

### Analysis of HERV-K expression

The expression of the HERV-K elements were analysed using SalmonTE tool [28]. Briefly, we built a human reference using RepBase fasta files and used fastq files from the Target ALS cohort to quantify the expression of repetitive elements. After quantification of the read counts, the differential expression analysis was performed by using DESeq2 package in R.

### Genotyping reference L1HS elements in whole genome sequencing data

WGS in BAM format aligned to Hg38 of 239 individuals from the Target ALS cohort were obtained from the New York Genome Center. This included 179 with ALS, 33 with ALSND and 27 NNC. L1HS elements were genotyped using the structural variant (SV) caller DELLY (https://github.com/dellytools/delly) and default parameters [29]. The SVs called by DELLY as deletions and that overlapped with co-ordinates of the 400 L1HS elements with a 5’UTR were extracted. The 70 L1HS detected as polymorphic were divided into intact and non-intact groups. The proportion of polymorphic intact and non-intact L1s with an IAF > 0.75 was compared using the prop.test in R studio.

### Comparison of location and sequence of L1HS elements

The coordinates of the 400 L1HS elements with a 5’UTR were split into those that were expressed (92 L1s) and those not expressed (308 L1s) in the four tissues analysed. These coordinates were intersected with those of introns in the UCSC genome browser (https://genome.ucsc.edu/) to determine the percentage of expressed and not expressed L1s that were present in introns. The sequence of the 400 L1HS elements were exported from the UCSC genome browser to Galaxy (https://usegalaxy.org.au/) and the sequences for the YY1, RUNX3 1 and 2 and SRY sites defined by L1Xplorer [30] were searched for to determine how many elements retained these sites. Fisher’s exact test was used to determine if the number of expressed or not expressed L1s for each defining characteristic were significantly different.

### Expression profiles of selected genes

Gene expression levels were calculated from the RNA-seq data mapped with STAR to the hg38 reference genome. Bam files were imported to the R studio workspace using *summarizeOverlaps* function from the package GenomicAlignments. Raw data were normalised by using median of ratios method implemented in the Deseq2 package. This method is based on dividing the counts by sample-specific size factors determined by median of gene counts relative to the geometric mean per gene. The normalised counts from all samples were averaged by genes and the expression values of specific genes in specific tissues were extracted for further analysis. The expression levels of the transcription factors (YY1 and RUNX3) were compared across the four tissues (ANOVA with Tukey adjustment for multiple comparisons). The mean expression of those genes containing an expressed L1 was compared to the expression of genes contain a not expressed L1 (Wilcoxon test). Correlation analysis of gene expression with L1 expression was performed using the Spearman rank correlation test.

## Results

### Tissue specific reduction in total L1 expression in ALS compared to controls

Proper L1 transcript expression, defined as occurring from L1HS elements with a 5’UTR and reads containing only L1 sequence or 3’run-on, was quantified using the L1EM software tool (https://github.com/FenyoLab/L1EM) [27] in RNA–sequencing data from the Target ALS cohort. Proper L1 expression was detected at a total of 92 loci across four tissues (motor cortex, frontal cortex, cerebellum and cervical spinal cord) from non-neurological controls (NNC) and individuals with ALS or ALS with other neurological disorder (ALSND). The L1 loci were divided into those that encode functional ORF1 and ORF2 proteins (intact) and those that do not (non-intact); 50 of the expressed L1 loci were intact elements and 42 non-intact L1s (Additional file 2: Table S2 for coordinates of expressed loci). The total number of expressed L1 loci was highest in the two cortices analysed (motor and frontal) followed by the cerebellum and the cervical spinal cord (Table 1). The mean number of L1 loci expressed in each individual again was highest in the cortices followed by the cerebellum and cervical spinal cord (Table 1).

**Table 1:** The number of different L1 loci expressed in each tissue

Tissue	Number of different L1 loci expressed		Mean number of L1 loci expressed			
			NNC		ALS/ALSND	
	Intact	Non-intact	Intact	Non-intact	Intact	Non-intact
Motor cortex	35	29	5.3 (0–10)	3.1 (0–7)	3.2 (0–16)	2.8 (0–17)
Frontal Cortex	36	32	4.3 (1–13)	4.9 (1–15)	2.5 (0–15)	2.9 (0–15)
Cerebellum	33	26	2.7 (0–10)	2.9 (0–7)	1.6 (0–9)	2.2 (0–8)
Spinal cord cervical	23	18	1.8 (0–4)	2.2 (0–5)	1.5 (0–7)	1.7 (0–9)

*NNC* non-neurological control, *ALS* amyotrophic lateral sclerosis, *ALSND* amyotrophic lateral sclerosis and other neurological disorder

**Fig. 1:**
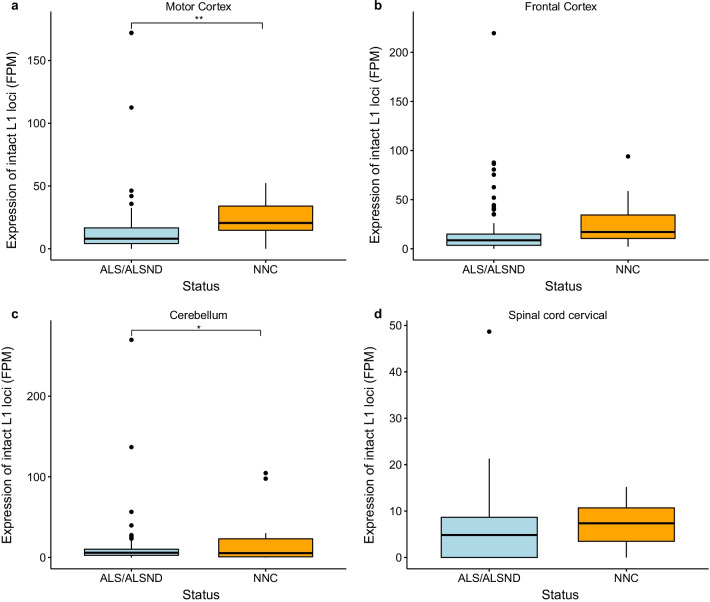
Expression of intact L1 loci is significantly lower in brain tissues of individuals with ALS or ALSND. **a**, **c** The total expression of intact L1 loci is significantly lower in the motor cortex (p = 0.006) and cerebellum (p = 0.04) of individuals with ALS or ALSND compared to controls. **b**, **d** There is no significant difference in the level of intact L1 expression in the frontal cortex (p = 0.06) cervical spinal cord of individuals with ALS or ALSND and controls (p = 0.51). Motor cortex- ALS/ALSND n = 92 and NNC n = 15, frontal cortex- ALS/ALSND n = 121 and NNC n = 15, cerebellum- ALS/ALSND n = 133 and NNC n = 14 and spinal cord cervical- ALS/ALSND n = 115 and NNC n = 13. *ALS* amyotrophic lateral sclerosis, *ALSND* amyotrophic lateral sclerosis and other neurological disorder, *NNC* non-neurological control, *FPM* fragments per million

**Fig. 2:**
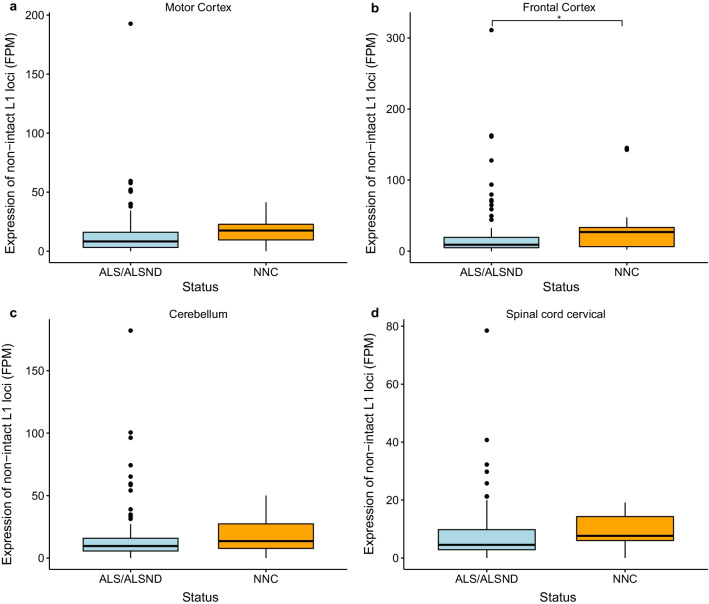
Expression of non-intact L1 loci is significantly lower in the frontal cortex of individuals with ALS or ALSND. **a** There was no significant difference in the level of non-intact L1 expression in the motor cortex of individuals with ALS or ALSND and controls (p = 0.78). **b** The total expression of non-intact L1 loci is significantly lower in the frontal cortex (p = 0.02) of individuals with ALS or ALSND compared to controls. **c**, **d** There is no significant difference in the level of non-intact L1 expression in the cerebellum (p = 0.10) or cervical spinal cord (p = 0.31) of individuals with ALS or ALSND and controls. Motor cortex-ALS/ALSND n = 92 and NNC n = 15, frontal cortex-ALS/ALSND n = 121 and NNC n = 15, cerebellum-ALS/ALSND n = 133 and NNC n = 14 and spinal cord cervical-ALS/ALSND n = 115 and NNC n = 13. *ALS* amyotrophic lateral sclerosis, *ALSND* amyotrophic lateral sclerosis and other neurological disorder, *NNC* non-neurological control, *FPM* fragments per million

The total expression of the intact L1 loci was significantly lower in the motor cortex (p = 0.006) and cerebellum (p = 0.02) of individuals with ALS or ALSND compared to controls, however there was no significant difference in the frontal cortex (p = 0.06) and cervical spinal cord (p = 0.51) (Fig. 1). There was no significant difference in the total expression of non-intact L1 loci in three of the tissues analysed (motor cortex, cerebellum and cervical spinal cord), however it was significantly reduced in the frontal cortex of individuals with ALS or ALSND (p = 0.04) (Fig. 2). This analysis excluded those outliers (more than 3 SD from the mean) when comparing the total expression showing that the general trend was a reduction in L1 expression in these regions in ALS or ALSND. However, there were a few individuals with ALS or ALSND in which L1 expression was massively increased, up to 25 times the average of intact elements and up to 15 times the average of non-intact elements depending on the tissue (Figs. 1 and 2). For a number of the ALS and ALSND individuals in the cohort, *C9orf72* expansion carrier status was known (MCX n = 79, FCX n = 104, CER n = 115 and SCC n = 99), therefore the level of intact and non-intact L1 expression was compared in those with and without the expansion and there was no significant difference identified (MCX p = 0.61 and p = 0.81, FCX p = 0.87 and p = 0.54, CER p = 0.88 and p = 0.48 and SCC p = 0.32 and p = 0.41). There were no individuals analysed in this study who were carriers of *SOD1*, *FUS* or *TARDBP* pathogenic variants. In some studies HERV-K expression has been reported to be significantly upregulated in ALS, therefore to determine if this was observed in the Target ALS dataset we compared HERV-K levels in the NNC to the individuals with ALS or ALSND. We identified four different HERV-K lineages that were expressed at a significantly lower level in the NNC with at least one lineage downregulated in each tissue (Additional file 3: Table S3).

### Specific L1 loci expression differs between individuals with ALS and controls

**Fig. 3:**
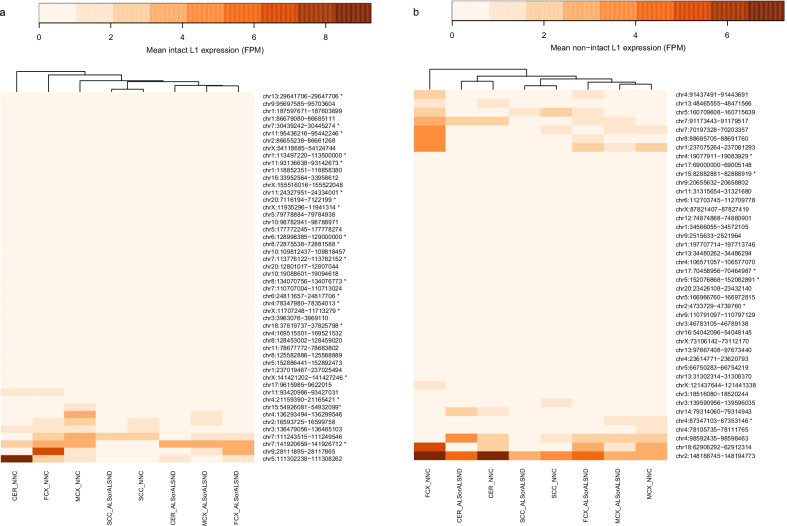
L1 loci specific expression across four tissues. **a** The mean expression of each of the 50 intact L1 loci across four tissues (motor cortex, frontal cortex, cerebellum and spinal cord cervical) of individuals with ALS or ALSND and controls. **b** The mean expression of each of the 42 non-intact L1 loci across four tissues (motor cortex, frontal cortex, cerebellum and spinal cord cervical) of individuals with ALS or ALSND and controls. Motor cortex-ALS/ALSND n = 92 and NNC n = 15, frontal cortex-ALS/ALSND n = 121 and NNC n = 15, cerebellum-ALS/ALSND n = 133 and NNC n = 14 and spinal cord cervical- ALS/ALSND n = 115 and NNC n = 13. *ALS* amyotrophic lateral sclerosis, *ALS* amyotrophic lateral sclerosis and other neurological disorder, *NNC* non-neurological control, *FPM* fragments per million, *polymorphic L1s

**Fig. 4:**
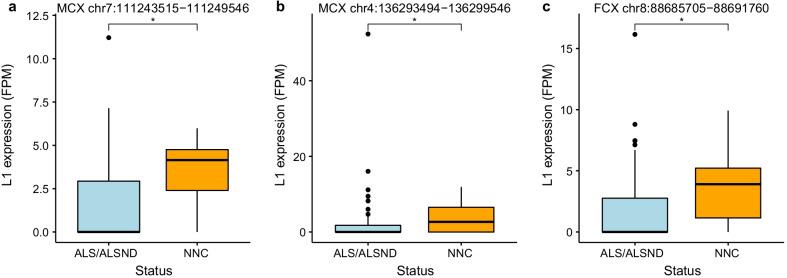
The expression of three individual L1 loci was significantly lower in individuals with ALS or ALSND. **a** The expression of the intact L1 at chr7:111,243,515–111,249,546 in the intron of the *IMMPL2* gene was significantly lower (p_adj_ = 0.012) in the motor cortex of individuals with ALS or ALSND compared to controls. **b** The expression of the intact L1 at chr4:136,293,494–136,299,546 was significantly lower (p_adj_ = 0.035) in the motor cortex of individuals with ALS or ALSND compared to controls. **c** The expression of the non-intact L1 at chr8:88,685,705–88,691,760 was significantly lower (p_adj_ = 0.013) in the frontal cortex of individuals with ALS or ALSND compared to controls. Wilcoxon test with Benjamini–Hochberg adjustment *p < 0.05, *MCX* motor cortex, *FCX* frontal cortex, *ALS* amyotrophic lateral sclerosis, *ALSND* amyotrophic lateral sclerosis and other neurological disorder, *NNC* non-neurological control, *FPM* fragments per million

Analysis of the mean expression from each L1 locus showed the majority of the L1 expression in this set was driven by a subset of elements for both the intact and non-intact L1s (Fig. 3). The heatmap in Fig. 3a shows the clustering of the three brain tissues (motor cortex, frontal cortex and cerebellum) of the controls (NNC) and clustering of the three brain regions of individuals with ALS or ALSND based on the expression profile of the intact L1s. The clustering of multiple regions from controls or ALS/ALSND is not observed for the non-intact L1 expression (Fig. 3b). To determine if differences in L1 expression were locus specific the expression of L1s (intact and non-intact) that was detected in > 25% of individuals in a particular tissue was compared. After adjusting for multiple comparisons three L1 loci were significantly reduced in individuals with ALS/ALSND compared to controls in a tissue specific manner (Fig. 4). The L1s at chr7:111,243,515–111,249,546 and chr4:136,293,494–136,299,546 are both intact L1s and were expressed at significantly lower levels in the motor cortex (p_adj_ = 0.012 and p_adj_ = 0.035 respectively) (Fig. 4a, b). The third L1 was a non-intact element at chr8:88,685,705–88,691,760 whose expression was significantly lower in the frontal cortex (p_adj_ = 0.013) (Fig. 4c). The L1 chr7:111,243,515–111,249,546 is located in an intron of the inner mitochondrial membrane peptide subunit 2 (*IMMP2L*) gene and the L1s at chr4:136,293,494–136,299,546 and chr8:88,685,705–88,691,760 are located in the introns of the long non-coding RNAs AC018680.1/lnc-PCDH18-4/ENST00000500324.2 and AC090578.1/RP11-586K2.1/ENST00000649573.1 respectively. Utilising the GTEx portal browser (https://www.gtexportal.org/home/) expression of *IMMP2L* was observed in all tissues analysed in the database, including several regions of the brain, however the expression of the long non-coding RNA AC018680.1 was restricted to the testis and AC090578.1 was expressed at very low levels in several tissues including the brain.

### Members of the L1HS subfamily are polymorphic for their presence/absence in the Target ALS cohort

Reference L1HS elements can be polymorphic for their presence or absence, therefore WGS available for 239 individuals from the Target ALS cohort was analysed to identify variation at the 400 L1HS elements with a 5’UTR. In this cohort 70 of these L1s were detected as polymorphic; 40 of which were intact (see Additional file 4: Table S4 for coordinates and insertion allele frequencies of each L1). Three of the polymorphic L1s identified in our analysis had been genotyped for their presence/absence using PCR amplification in our previous study [25]. The intact L1s had lower insertion allele frequencies (IAF) compared to the non-intact loci, for example non-intact L1s had a significantly higher proportion of L1s with an IAF > 0.75 compare to intact loci (50% vs 22.5%, p = 0.03) (Additional file 6: Figure S2). The number of individuals with genotypes available was too low for a case control analysis to determine if the variation of these elements was contributing to disease risk.

### A higher proportion of expressed L1 loci are present in introns compared to those not expressed

**Fig. 5:**
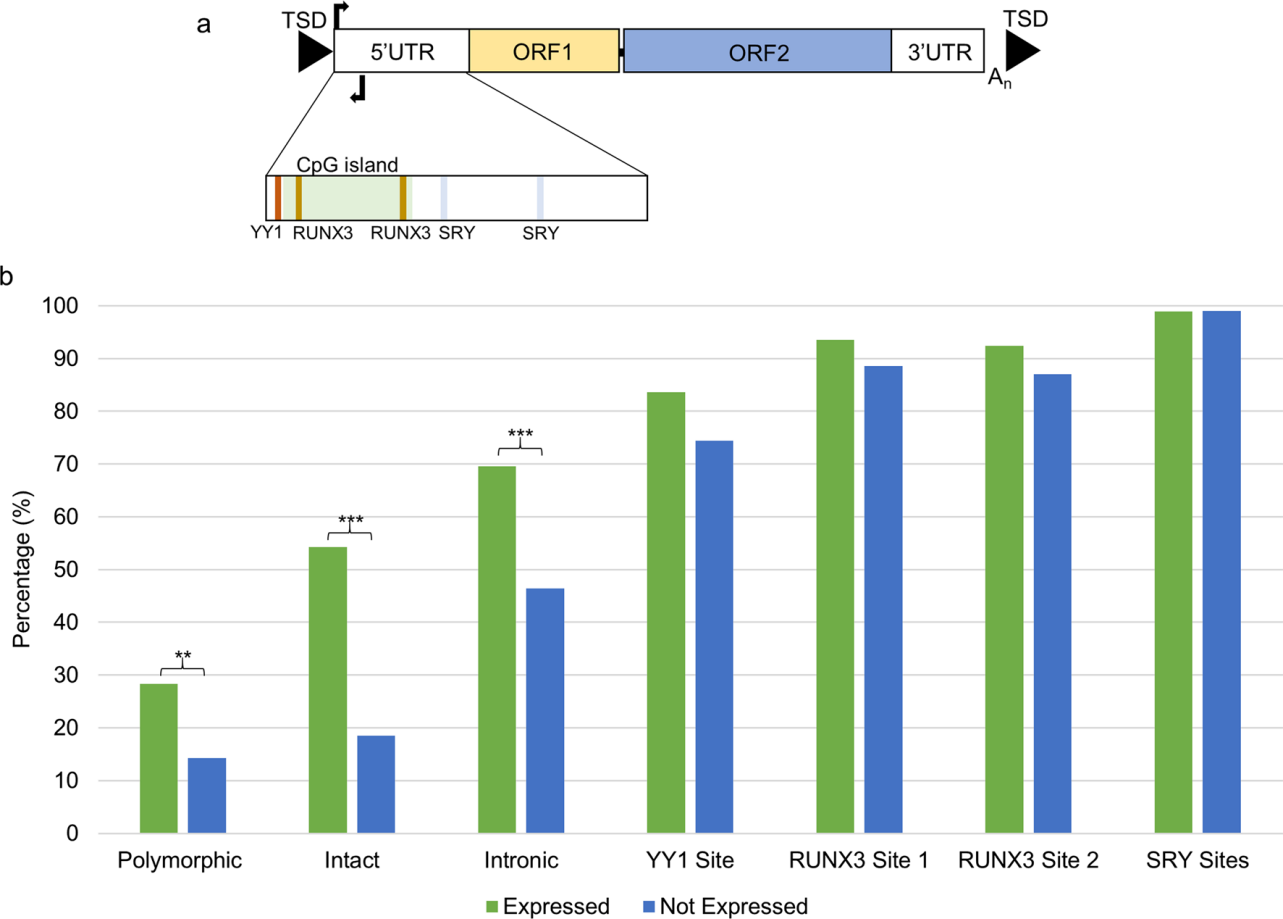
A higher percentage of expressed L1 loci are polymorphic and intronic. **a** The structure of a functional full length L1 consists of a 5’UTR, containing the endogenous L1 promoter and an antisense promoter, two ORFs encoding proteins required for retrotransposition and a 3’UTR, is ~ 6 kb in length and insertions are usually flanked by TSDs. The expanded 5’UTR cartoon illustrates features of this region, which include a CpG island and the following transcription factor binding sites YY1, RUNX3 and SRY. **b** The percentage of expressed L1s and not expressed L1s that have the following defining characteristics: polymorphic, intact, intronic and the presence of the following conserved transcription factor binding sites YY1, RUNX3 and SRY. **p < 0.01, ***p < 0.001, *TSDs* target site duplications, *UTR* untranslated region, *ORF* open reading frame

**Fig. 6:**
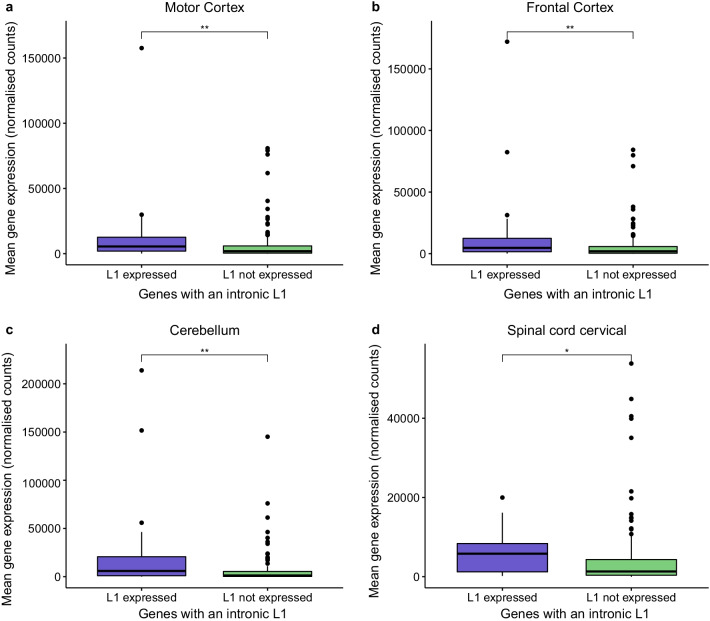
The mean expression of genes with a fixed intronic L1 that is expressed is higher than those genes with a fixed intronic L1 that is not expressed. **a**–**d** The mean expression of genes with a fixed (not polymorphic) intronic L1 was quantified from the RNA-sequencing data across the four tissues analysed for the L1 expression (motor cortex, frontal cortex, cerebellum and cervical spinal cord). The mean expression of those genes containing an expressed L1 was significantly higher than those containing a L1 that is not expressed. Wilcoxon test, *p < 0.05, **p < 0.01 and ***p < 0.001

To determine if the L1s with 5’UTRs (structure shown in Fig. 5a) that were expressed in the tissues analysed harboured different properties to those not expressed several characteristics were compared. Of the expressed L1s 28.3% were polymorphic for their presence or absence compared to 14.3% of those not expressed (p = 0.002), 54.5% were intact compared 18.5% (p = 0.00001) and 69.6% were located in introns compared to 46.4% (p = 0.0001) (Fig. 5b). There was no significant difference in the presence of conserved YY1, RUNX3 and SRY transcription factor sites between those expressed L1s and those not expressed (Fig. 5). The levels of RUNX3 and YY1 expression was compared across the four tissues (Additional file 7: Figure S3). The expression of RUNX3 was significantly higher in the cervical spinal cord than the three brain regions (Additional file 7: Figure S3a) and YY1 levels was significantly higher in the cerebellum compared to the other three tissues (Additional file 7: Figure S3b). The intronic location of the L1s was one of the predominant characteristics that differed between the two groups (expressed vs not expressed). Therefore, the mean level of expression of those genes containing a fixed L1 were compared for each tissue to determine if the level of expression differed between those genes harbouring an expressed L1 compared to those genes with an L1 that was not expressed. The genes that harboured an expressed fixed intronic L1 had a significantly higher mean expression than those with a fixed intronic L1 that was not expressed in all four tissues (motor cortex p = 0.001, frontal cortex p = 0.015, cerebellum p = 0.0008 and cervical spinal cord p = 0.007) (Fig. 6). We then compared the mean expression of the gene and the L1 located in its intron to determine if there was a correlation between these two variables. In the motor cortex, frontal cortex and cerebellum there was a significant moderate positive correlation between the mean gene and L1 expression levels (motor cortex ρ = 0.63 p = 0.0006, frontal cortex ρ = 0.46 p = 0.01 and cerebellum ρ = 0.52 p = 0.01) (Additional file 8: Figure S4a-c). The weaker positive correlation observed between the two variables in the cervical spinal cord was not significant (ρ = 0.39 p = 0.07) (Additional file 8: Figure S4d). This analysis focused on the proper expression of L1s, however passive transcription of these elements can occur particularly for those located in introns. Of those L1HS elements that have a 5’UTR 207 were located in the intron of a Gencode transcript. In the motor cortex 46 of the intronic L1s demonstrated proper expression and of the remaining 161 intronic L1s there was evidence of passive transcription at 75 loci.

**Fig. 7:**
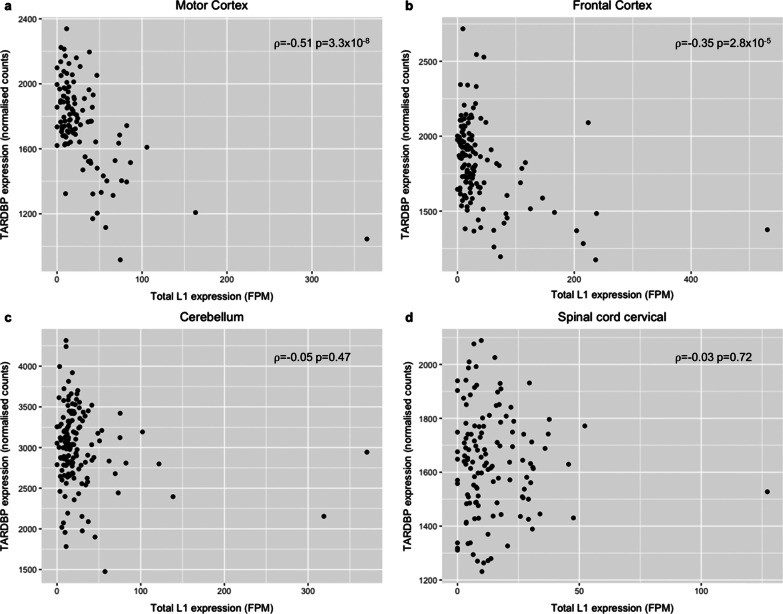
A significant negative correlation in the expression of TARDBP and L1 expression in the motor and frontal cortices. **a** There was a significant negative correlation between the expression of the major transcript of TARDBP (ENST00000240185.8) and total L1 expression in the motor cortex (ρ = -0.51 p = 3.3 × 10^–8^). **b** There was a significant negative correlation between the expression of the major transcript of TARDBP (ENST00000240185.8) and total L1 expression in the frontal cortex (ρ =  − 0.35 p = 0.47). **c** There was no significant correlation between the expression of the major transcript of TARDBP (ENST00000240185.8) and total L1 expression in the cerebellum (ρ =  − 0.05 p = 0.47). **d** There was no significant correlation between the expression of the major transcript of TARDBP (ENST00000240185.8) and total L1 expression in the cervical spinal cord (ρ =  − 0.03 p = 0.72). ρ = Spearman’s rank correlation coefficient

It has been reported previously that individuals with high levels of retrotransposon expression have lower levels of *TARDBP* and that TDP-43 silences their expression through binding to retrotransposons transcripts [11]. In addition, enhanced chromatin accessibility over L1s is associated with nuclear loss of TDP-43 [15]. Therefore, we compared the expression of the major transcript of *TARDBP* (ENST00000240185.8) with total L1 expression for each individual. In the motor and frontal cortices there was a significant negative correlation of *TARDBP* and L1 expression (ρ =  − 0.51 p = 3.3 × 10^–8^ and ρ =  − 0.35 p = 2.8 × 10^–5^ respectively) (Fig. 7a, b). There was no correlation of *TARDBP* and L1 levels in the cerebellum (ρ =  − 0.05 p = 0.47) and cervical spinal cord (ρ =  − 0.03 p = 0.72) (Fig. 7c, d).

## Discussion

The expression of transposable elements, including several families of retrotransposons, has been characterised in ALS identifying an activation and upregulation of these elements in the CNS of individuals with the disease [6, 7, 10, 11]. There has been a limited number of studies on the specific loci of retrotransposons that are expressed. The predominant focus in ALS has been characterising loci specific HERV expression with differences observed in whether there is a significant upregulation or not of HERV loci [6, 7, 9]. One recent study profiling the loci specific expression of HERVs identified one HERV locus (HML6_3p21.31c) that was consistently upregulated in the motor cortex and cerebellum of individuals with ALS compared to controls [31]. Therefore, we sought to address the question of L1 loci specific expression in ALS utilising RNA-sequencing data from multiple tissues obtained from the New York Genome Center Target ALS cohort. The L1EM tool was used to profile L1HS expression in a total of 518 samples from the following tissues; motor cortex (107), frontal cortex (136), cerebellum (147) and cervical spinal cord (128). L1s are the only autonomous retrotransposons that have retained the ability to retrotranspose in the human genome and generate new insertions. Further, it is only a small number of these L1s (RC-L1s) that can mobilise, therefore identifying the specific L1 loci that are expressed will inform if those L1s encode functional ORF1 and 2 proteins and could potentially retrotranspose. Here we identified a general reduction in the total expression of intact L1s (those encoding functional proteins) in two brain regions (motor cortex and cerebellum) of those individuals with ALS compared to controls. This could suggest that L1 expression from those intact elements is potentially beneficial as it is higher in individuals not suffering from a neurological condition. For example L1s have been implicated in memory and learning as long term memory formation was impaired in mouse models using L1 inhibitors [32]. However, there is the caveat that the window of L1 expression captured in post-mortem tissues may not reflect L1 expression throughout disease development. This observed reduction in L1 expression contrasts with studies addressing retrotransposons at the subfamily level, for example L1HS, that identified an upregulation of these elements in ALS [11]. We analysed a very specific subset of L1s which could account for the different results observed in our study compared to previous publications. However, there were a handful of individuals with ALS or ALSND in our study that showed a large upregulation of L1s compared to the rest of the cohort which may be line with Tam et al. [11] who demonstrated that it is a subset of ALS that is characterised by the activation of retrotransposons rather than being a feature in all cases of the disease.

When comparing the pattern of expression from specific intact L1 loci we noted that the three brain regions of the controls clustered together as did those of the individuals with ALS or ALSND (Fig. 3a). However, this was not the case when comparing the non-intact L1. This suggests that the pattern of intact L1 loci expression could be disease related, for at least, in tissues related to the brain. There are only a limited number of studies characterising human L1 loci specific expression for comparison with our study, furthermore the majority of the literature describes their expression in cell lines [33–36]. In the latter, L1 expression is often restricted to a small number of elements and can be cell line dependent [33, 36]. Mckerrow et al., who developed the L1EM tool used in this analysis, analysed L1 expression in over 120 datasets that included cell lines and tissues from the ENCODE database [27]. The authors showed L1 expression in multiple cancer cell lines, embryonic stem cells and several lines derived from the embryonic cell lineage with limited expression detected in the tissues analysed from the ENCODE database. A sizable proportion of the intact L1 expression (17%) of all the samples in this previous publication was from a single L1 locus at chr22:28,663,283–28,669,315, which is responsible for the most somatic L1 insertions in tumours [22, 37]. Interestingly we did not detect any expression from this L1 locus in the samples analysed here, providing evidence for tissue specific expression of these elements. This could be related to regulation of L1s by CpG methylation, for example a recent study showed that this L1 located on chr22 is hypomethylated in the liver compared to the heart and hippocampus [38]. Our previous work analysing the methylation status of 6 highly active RC-L1s in the motor cortex identified a reduction in the DNA methylation over these elements in ALS brains compared to controls [25]. Five of these elements located in the reference genome were also analysed as part of this study quantifying their expression levels and found that four of these L1s were expressed, however they were not part of the group of L1s responsible for the majority of the expression observed.

One of the significant characteristics of the 92 expressed L1s compared to the 308 L1s not expressed was the percentage that were in introns (69.6% vs 46.4%). We demonstrated that the level of expression of genes with an intronic expressed L1 was on average significantly higher than that of genes with an intronic L1 that was not expressed in all four tissues analysed (Fig. 6) and the level of the expressed L1 significantly positively correlated with the expression of the gene in the three brain regions analysed (Additional file 7: Figure S3). This is in agreement with the analysis by Philippe et al. who also showed that genes with an expressed L1 had higher expression than those genes with a L1 which was not expressed [36]. Although this suggests that an L1 located in the intron of a gene, particularly if that gene is highly expressed, is more likely to be expressed in that tissue it is not a prerequisite for L1 expression as there were 28 intergenic L1s that were expressed in our study. In addition to comparing L1 expression with the expression of the gene in which they were located we also compared L1 expression with that of *TARDBP*. Previous studies have shown that TDP-43 (encoded by the *TARDBP* gene) regulates the expression of retrotransposons [11, 14, 15] and that the subset of ALS characterised by retrotransposons activation also has lower levels of *TARDBP* (11). We identified that in the motor and frontal cortices lower levels of *TARDBP* is significantly correlated with increased L1 expression (Fig. 7). We were limited to analysing *TARDBP* expression to look for potential relationships with L1 expression as opposed to characterising TDP-43 dysfunction in terms of protein aggregation or mislocalisation due to the type of data available.

## Conclusions

We have completed the first study of L1 loci specific expression in ALS identifying an overall reduction in total intact L1 expression in two brain regions of those individuals with ALS and found that individuals clustered by diagnosis when comparing the pattern of intact L1 loci expression. Although in general L1 expression levels were reduced in ALS there were a small number of individuals overexpressing L1s in comparison to the rest of the cohort. This study highlights the subset of L1s that are responsible for the majority of expression in the four tissues analysed and the importance of understanding the regulation of these elements in the brain. Further investigation into the regulation of L1s in individuals that are extreme outliers in terms of their L1 expression profile would provide useful data in terms of how these elements become activated in certain individuals.

## Supplementary Information

**Additional file 1: Table S1.** Covariates used in the statistical analysis comparing total intact and non-intact L1 expression for each tissue.

**Additional file 2: Table S2.** Coordinates of intact and non-intact expressed L1 loci.

**Additional file 3: Table S3.** HERV-K lineages that were significantly differentially expressed in the motor cortex, frontal cortex, cerebellum and cervical spinal cord between NNC and ALS/ALSND.

**Additional file 4: Table S4.** Coordinates and insertion allele frequencies of polymorphic intact and non-intact L1s identified in the Target ALS cohort.

**Additional file 5: Figure S1.** Flow chart outlining analysis of RNA and WGS sequencing data.

**Additional file 6: Figure S2.** Comparison of the insertion allele frequency of polymorphic intact and non-intact L1s detected in the Target ALS cohort. The insertion allele frequency was calculated for the 70 polymorphic L1HS elements with a 5’UTR that were detected in the Target ALS cohort. The non-intact polymorphic L1s were more common in the population than the intact elements and the proportion of non-intact elements with an IAF > 0.75 was significantly higher than the intact L1s (p = 0.03) (prop.test).

**Additional file 7: Figure S3.** Comparison of RUNX3 and YY1 gene expression in the motor cortex, frontal cortex, cerebellum and cervical spinal cord. a – The expression of the transcription factor RUNX3 was significantly higher in the cervical spinal cord compared to the three brain tissues analysed (motor cortex, frontal cortex and cerebellum). b – The expression of the transcription factor YY1 was significantly higher in the cerebellum compared to the three other tissues analysed (motor cortex, frontal cortex and cervical spinal cord). ANOVA with Tukey adjustment for multiple comparisons ***p < 0.001 and ****p > 0.0001.

**Additional file 8: Figure S4.** The relationship between the mean gene expression and L1 expression when located in its intron in four different tissues. a – There was a significant positive correlation between the mean gene and L1 expression located within its intron in the motor cortex (ρ = 0.63 p = 0.0006) (number of gene and L1 pairs analysed = 27). b – There was a significant positive correlation between the mean gene and L1 expression located within its intron in the frontal cortex (ρ = 0.46 p = 0.01) (number of gene and L1 pairs analysed = 31). c – There was a significant positive correlation between the mean gene and L1 expression located within its intron in the cerebellum (ρ = 0.52 p = 0.01) (number of gene and L1 pairs analysed = 23). d – There was no significant correlation between the mean gene and L1 expression located within its intron in the cervical spinal cord (ρ = 0.39 p = 0.07) (number of gene and L1 pairs analysed = 22). ρ = Spearman’s rank correlation coefficient.

## Data Availability

The sequencing (RNA and WGS) analysed in this study from the Target ALS cohort were obtained upon application to the New York Genome Center and data requests can be made by completing a genetic data request form at ALSData@nygenome.org.

## Abbreviations

ALS: Amyotrophic lateral sclerosis
ALSND: Amyotrophic lateral sclerosis and other neurological disorder
CER: Cerebellum
ENCODE: Encyclopedia of DNA elements
FCX: Frontal cortex
IAF: Insertion allele frequency
L1/L1HS: Long interspersed element-1/long interspersed element-1 Homo sapiens
MCX: Motor cortex
NNC: Non-neurological control
ORF: Open reading frame
RCL1: Retrotransposition competent L1
SCC: Spinal cord cervical
SV: Structural variant
UTR: Untranslated region
