# Supplementary material for: Locus specific reduction of L1 expression in the cortices of individuals with amyotrophic lateral sclerosis
Source: Mol Brain. 2022 Mar 28;15:25. doi: 10.1186/s13041-022-00914-x (PMC8961898; doi:10.1186/s13041-022-00914-x)
Supplement: Supplementary file 3 — Additional file 3: Table S3. HERV-K lineages that were significantly differentially expressed in the motor cortex, frontal cortex, cerebellum and cervical spinal cord between NNC and ALS/ALSND. [file 13041_2022_914_MOESM3_ESM.docx]

Additional Table 3: HERVK lineages whose expression was significantly different in NNC compared to ALS/ALSND.

| Tissue | HERVK | Log2FC | Adjusted pvalue |
| --- | --- | --- | --- |
| Motor cortex | HERVK22I | -0.09 | 0.03 |
|  | HERVK9I | -0.11 | 0.04 |
|  | HERVKC4 | -0.55 | 0.03 |
|  | HERVK14I | -0.27 | 0.02 |
| Frontal Cortex | HERVK14I | -0.26 | 0.04 |
| Cerebellum | HERVK9I | -0.13 | 0.03 |
|  | HERVKC4 | -0.36 | 0.03 |
|  | HERVK14I | -0.25 | 0.02 |
| Cervical spinal cord | HERVKC4 | -1.19 | 9.83x10^-5^ |
|  | HERVK9I | -0.19 | 0.005 |
