# Supplementary figures and images for: Locus specific reduction of L1 expression in the cortices of individuals with amyotrophic lateral sclerosis
Source: Mol Brain. 2022 Mar 28;15:25. doi: 10.1186/s13041-022-00914-x (PMC8961898; doi:10.1186/s13041-022-00914-x)

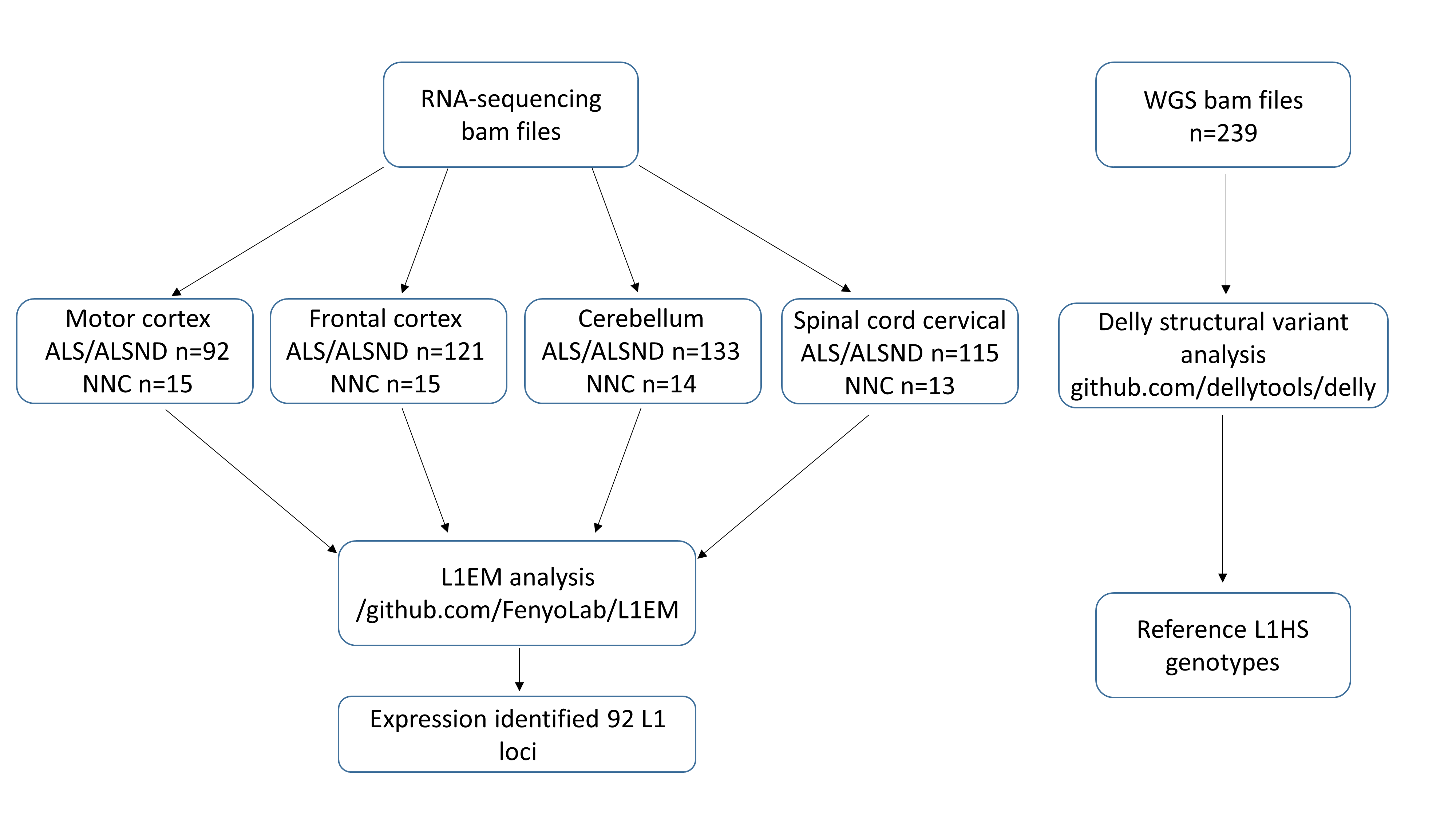

Supplement: Supplementary file 5 — Additional file 5: Figure S1. Flow chart outlining analysis of RNA and WGS sequencing data. [file 13041_2022_914_MOESM5_ESM.tif]

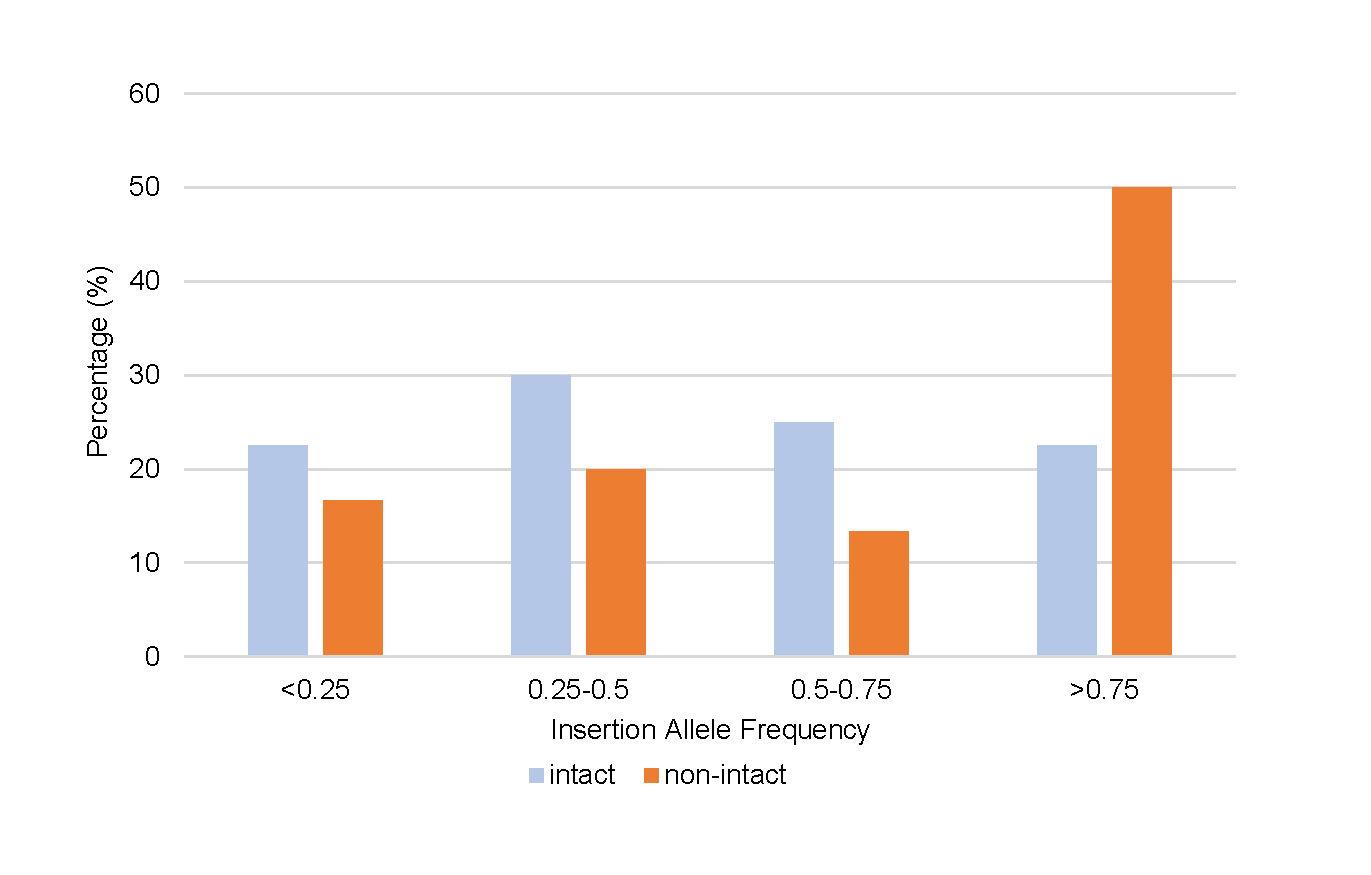

Supplement: Supplementary file 6 — Additional file 6: Figure S2. Comparison of the insertion allele frequency of polymorphic intact and non-intact L1s detected in the Target ALS cohort. The insertion allele frequency was calculated for the 70 polymorphic L1HS elements with a 5’UTR that were detected in the Target ALS cohort. The non-intact polymorphic L1s were more common in the population than the intact elements and the proportion of non-intact elements with an IAF > 0.75 was significantly higher than the intact L1s (p = 0.03) (prop.test). [file 13041_2022_914_MOESM6_ESM.tiff]

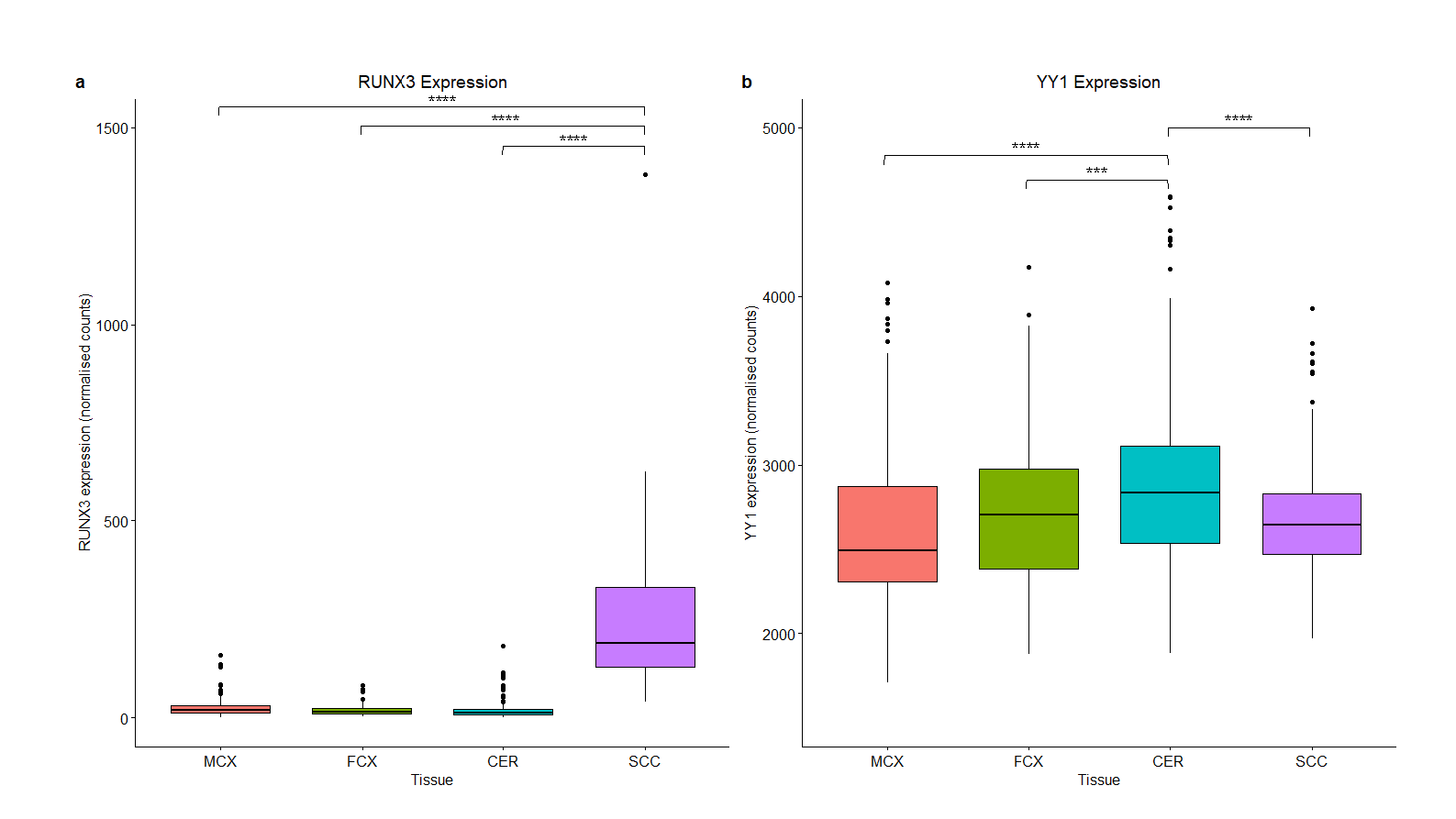

Supplement: Supplementary file 7 — Additional file 7: Figure S3. Comparison of RUNX3 and YY1 gene expression in the motor cortex, frontal cortex, cerebellum and cervical spinal cord. a – The expression of the transcription factor RUNX3 was significantly higher in the cervical spinal cord compared to the three brain tissues analysed (motor cortex, frontal cortex and cerebellum). b – The expression of the transcription factor YY1 was significantly higher in the cerebellum compared to the three other tissues analysed (motor cortex, frontal cortex and cervical spinal cord). ANOVA with Tukey adjustment for multiple comparisons ***p < 0.001 and ****p > 0.0001. [file 13041_2022_914_MOESM7_ESM.tiff]

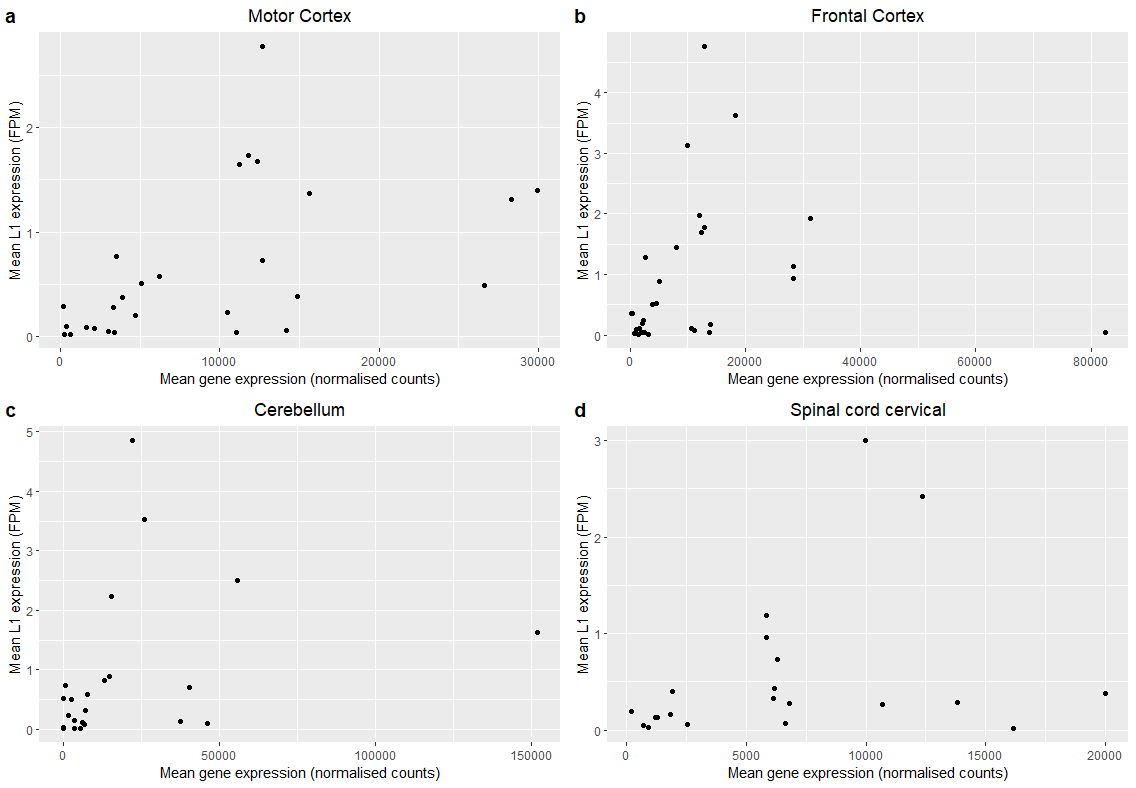

Supplement: Supplementary file 8 — Additional file 8: Figure S4. The relationship between the mean gene expression and L1 expression when located in its intron in four different tissues. a – There was a significant positive correlation between the mean gene and L1 expression located within its intron in the motor cortex (ρ = 0.63 p = 0.0006) (number of gene and L1 pairs analysed = 27). b – There was a significant positive correlation between the mean gene and L1 expression located within its intron in the frontal cortex (ρ = 0.46 p = 0.01) (number of gene and L1 pairs analysed = 31). c – There was a significant positive correlation between the mean gene and L1 expression located within its intron in the cerebellum (ρ = 0.52 p = 0.01) (number of gene and L1 pairs analysed = 23). d – There was no significant correlation between the mean gene and L1 expression located within its intron in the cervical spinal cord (ρ = 0.39 p = 0.07) (number of gene and L1 pairs analysed = 22). ρ = Spearman’s rank correlation coefficient. [file 13041_2022_914_MOESM8_ESM.tiff]
